# Improvement in Zebrafish with Diabetes and Alzheimer's Disease Treated with Pasteurized Akkermansia muciniphila

**DOI:** 10.1128/spectrum.00849-23

**Published:** 2023-05-16

**Authors:** Linkai Qu, Fan Liu, Yimeng Fang, Lei Wang, Haojie Chen, Qinsi Yang, Hao Dong, Libo Jin, Wei Wu, Da Sun

**Affiliations:** a Institute of Life Sciences & Biomedical Collaborative Innovation Center of Zhejiang Province, Wenzhou University, Wenzhou, China; b College of Life Sciences, Jilin Agricultural University, Changchun, China; c Wenzhou Institute, University of Chinese Academy of Sciences, Wenzhou, China; d Key Laboratory for Biorheological Science and Technology of Ministry of Education, State and Local Joint Engineering Laboratory for Vascular Implants, Bioengineering College of Chongqing University, Chongqing, China; University of Arkansas for Medical Sciences

**Keywords:** *Akkermansia muciniphila*, diabetes, Alzheimer's disease, behavior, intestinal microorganism, zebrafish

## Abstract

Diabetes and Alzheimer's disease (AD) are associated with specific changes in the composition of the intestinal flora. Studies have shown that the supplementation with pasteurized Akkermansia muciniphila has therapeutic and preventive effects on diabetes. However, it is not clear whether there is any association with improvement in and prevention of Alzheimer's disease and diabetes with Alzheimer's disease. Here, we found that pasteurized Akkermansia muciniphila can significantly improve the blood glucose, body mass index, and diabetes indexes of zebrafish with diabetes mellitus complicated with Alzheimer's disease and also alleviate the related indexes of Alzheimer's disease. The memory, anxiety, aggression, and social preference behavior of zebrafish with combined type 2 diabetes mellitus (T2DM) and Alzheimer’s disease (TA zebrafish) were significantly improved after pasteurized Akkermansia muciniphila treatment. Moreover, we examined the preventive effect of pasteurized Akkermansia muciniphila on diabetes mellitus complicated with Alzheimer's disease. The results showed that the zebrafish in the prevention group were better in terms of biochemical index and behavior than the zebrafish in the treatment group. These findings provide new ideas for the prevention and treatment of diabetes mellitus complicated with Alzheimer's disease.

**IMPORTANCE** The interaction between intestinal microflora and host affects the progression of diabetes and Alzheimer's disease. As a recognized next-generation probiotic, Akkermansia muciniphila has been shown to play a key role in the progression of diabetes and Alzheimer's disease, but whether *A. muciniphila* can improve diabetes complicated with Alzheimer's disease and its potential mechanism are unclear. In this study, a new zebrafish model of diabetes mellitus complicated with Alzheimer's disease was established, and the effect of Akkermansia muciniphila on diabetes mellitus complicated with Alzheimer's disease is discussed. The results showed that Akkermansia muciniphila after pasteurization significantly improved and prevented diabetes mellitus complicated with Alzheimer's disease. Treatment with pasteurized Akkermansia muciniphila improved the memory, social preference, and aggressive and anxiety behavior of TA zebrafish and alleviated the pathological characteristics of T2DM and AD. These results provide a new prospect for probiotics in the treatment of diabetes and Alzheimer's disease.

## INTRODUCTION

Diabetes is a metabolic syndrome characterized by persistent chronic hyperglycemia. In 2019, there were about 463 million confirmed and undiagnosed diabetes cases worldwide, and they are projected to reach 700 million by 2045 (1). In addition to its direct harm to human health, diabetes is also associated with significant indirect complications and burdens to family members and society.

Alzheimer's disease (AD), a common neurodegenerative disease and complication of diabetes, significantly impacts patients' psychological and social well-being. It is estimated to have affected about 57.4 million people worldwide by 2019 and is projected to affect about 152.8 million by 2050 (2). A meta-analysis revealed that diabetes patients are twice as likely to develop memory impairment and dementia as those without diabetes with advancing age. There is evidence indicating that AD is also a metabolic disease and that, in healthy brains, amyloid-β (Aβ) is rapidly cleared and maintained at a low concentration (3, 4). Impaired Aβ homeostasis may cause its accumulation in the brain, which may promote the development of AD. Thus, AD is also known as “type 3 diabetes.” In addition to its negative effect on the patient’s mental health, long-term diabetes also causes insulin resistance (IR) in the brain, accelerates the formation of AD-associated brain pathology, impairs memory and cognition, and may lead to abnormal behavior, such as memory loss, anxiety, aggression, and social disorders (5).

**FIG 1:**
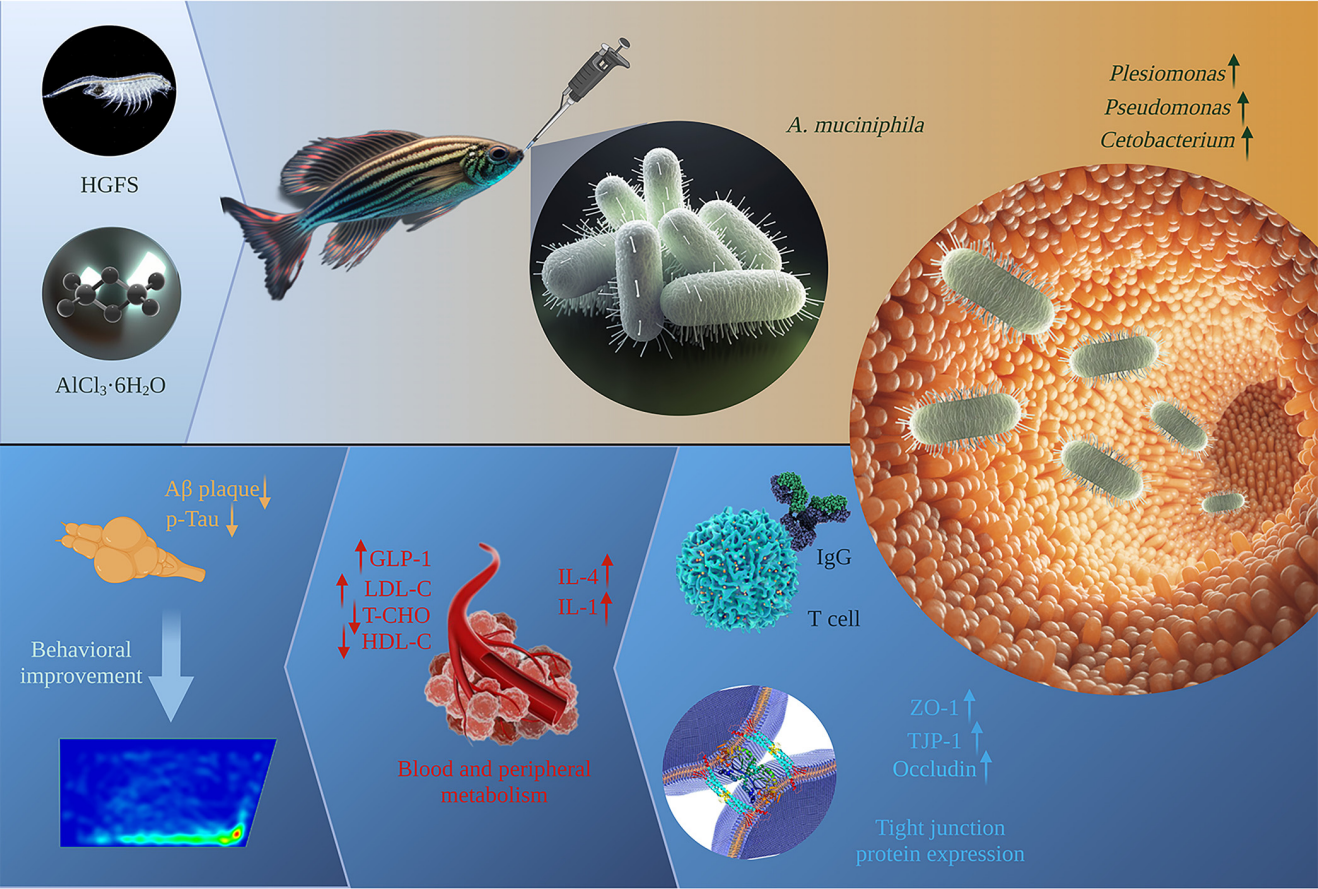
Schematic illustration of the model construction process and the mechanism of treatment and prevention of T2DM with AD in *A. muciniphila*.

Intestinal microorganisms living in the intestine are important to the pathology of diabetes mellitus (DM) and Alzheimer's disease (AD), and the levels of microorganisms such as Akkermansia muciniphila are significantly reduced in patients with DM and AD. Imbalances in intestinal microflora not only disrupt the integrity of the intestinal barrier but can also trigger intestinal metabolic disorders and chronic inflammation, which may cause bacteria and their metabolites to enter the circulatory system (6). Such intestinal barrier breaches also disrupt IR and glucose metabolism and may trigger neuron death, thereby injuring many organs, including the brain (7, 8). Ultimately, these effects may promote the occurrence of diseases like type 2 diabetes mellitus (T2DM) and AD. Moreover, *A. muciniphila* supplementation can partially alleviate T2DM and AD, restore intestinal barrier function, improve fat metabolism, glucose content, and IR, and delay the occurrence of brain lesions (9–11). It is reported that pasteurized *A. muciniphila* is a less extreme treatment that limits the degeneration of its cellular components, can partially or completely retain its beneficial effects, and is better than live *A. muciniphila* (12). However, it is unclear if it improves DM in the context of AD. To assess this possibility, we used zebrafish as an animal model. Zebrafish genes have a high similarity (up to 87%) to human genes (13). Moreover, the development of the cardiovascular and nervous systems of zebrafish is very similar to that of humans. Zebrafish are widely used to model human diseases, including DM (14), its complications (15, 16), and AD (17). After the successful establishment of the model, slightly modified based on the method described by Collymore et al. (18), zebrafish were given intragastric administration of *A. muciniphila*. We assessed if pasteurized *A. muciniphila* improves DM complications in the context of AD by monitoring blood glucose levels, biochemical indexes, and the level of intestinal microorganisms in adult zebrafish. The study design is summarized in Fig. 1.

## RESULTS

### A high-glucose fairy shrimp diet and AlCl_3_·6H_2_O trigger a phenotype of T2DM with AD in zebrafish.

**FIG 2:**
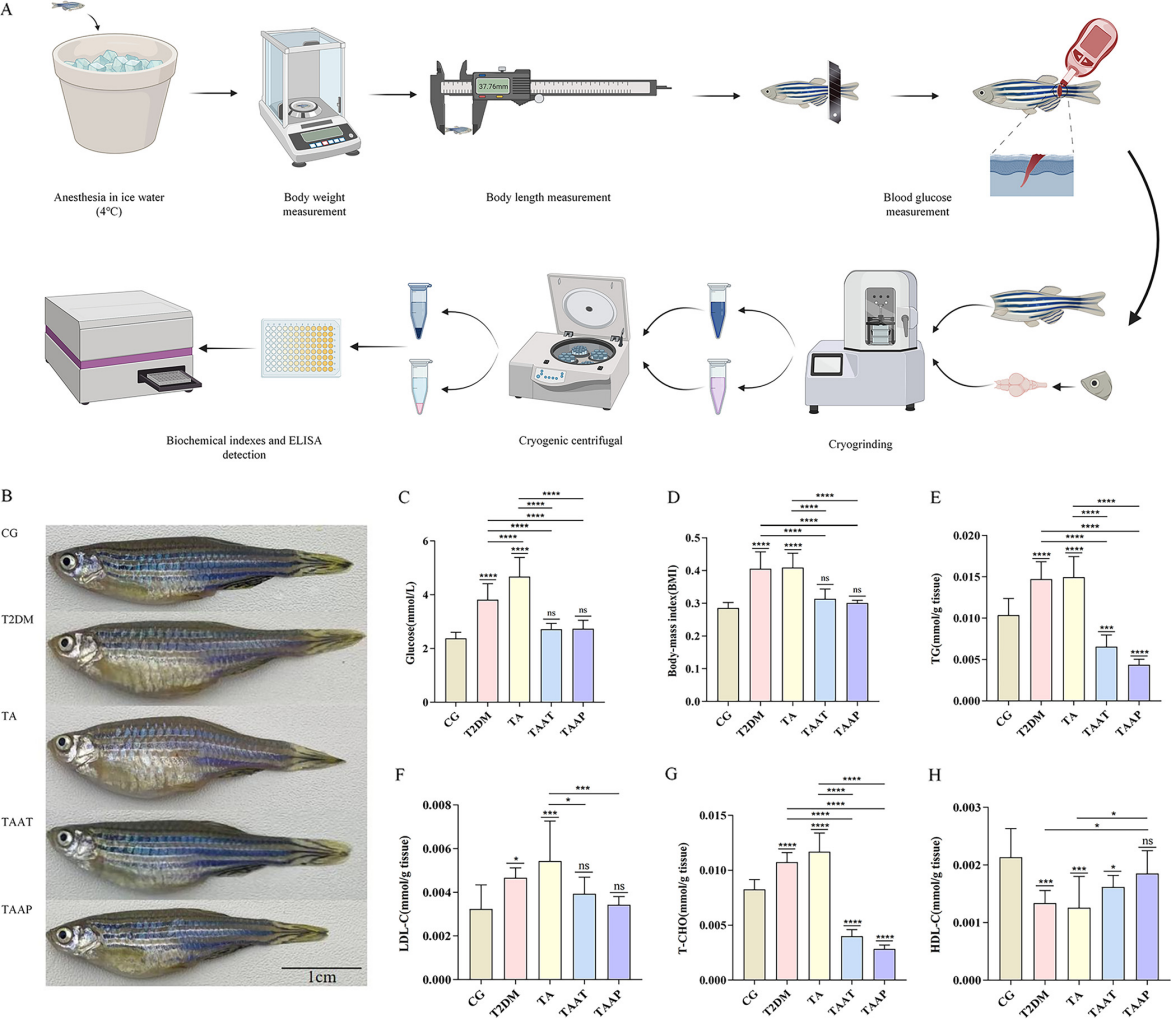
Whole-body tissue examination in different groups of zebrafish. (A) Flow chart of blood glucose, BMI, and biochemical indexes of zebrafish; (B) morphology of zebrafish before and after treatment; (C) level of glucose (*n* = 20 per group); (D) BMI (*n* = 20 per group); (E) triglyceride (TG) (*n* = 10 per group); (F) low-density lipoprotein (LDL-C) (*n* = 10 per group); (G) total cholesterol (T-CHO) (*n* = 10 per group); (H) high-density lipoprotein cholesterol (HDL-C) (*n* = 10 per group). Data are expressed as the mean ± SEM and were analyzed by one-way analysis of variance (ANOVA), followed by Tukey's *post hoc* test. Significance was defined as follows versus control groups (*n* = 20 per group): *, *P* < 0.05; **, *P* < 0.01; ***, *P* < 0.001; and ****, *P* < 0.0001.

**FIG 3:**
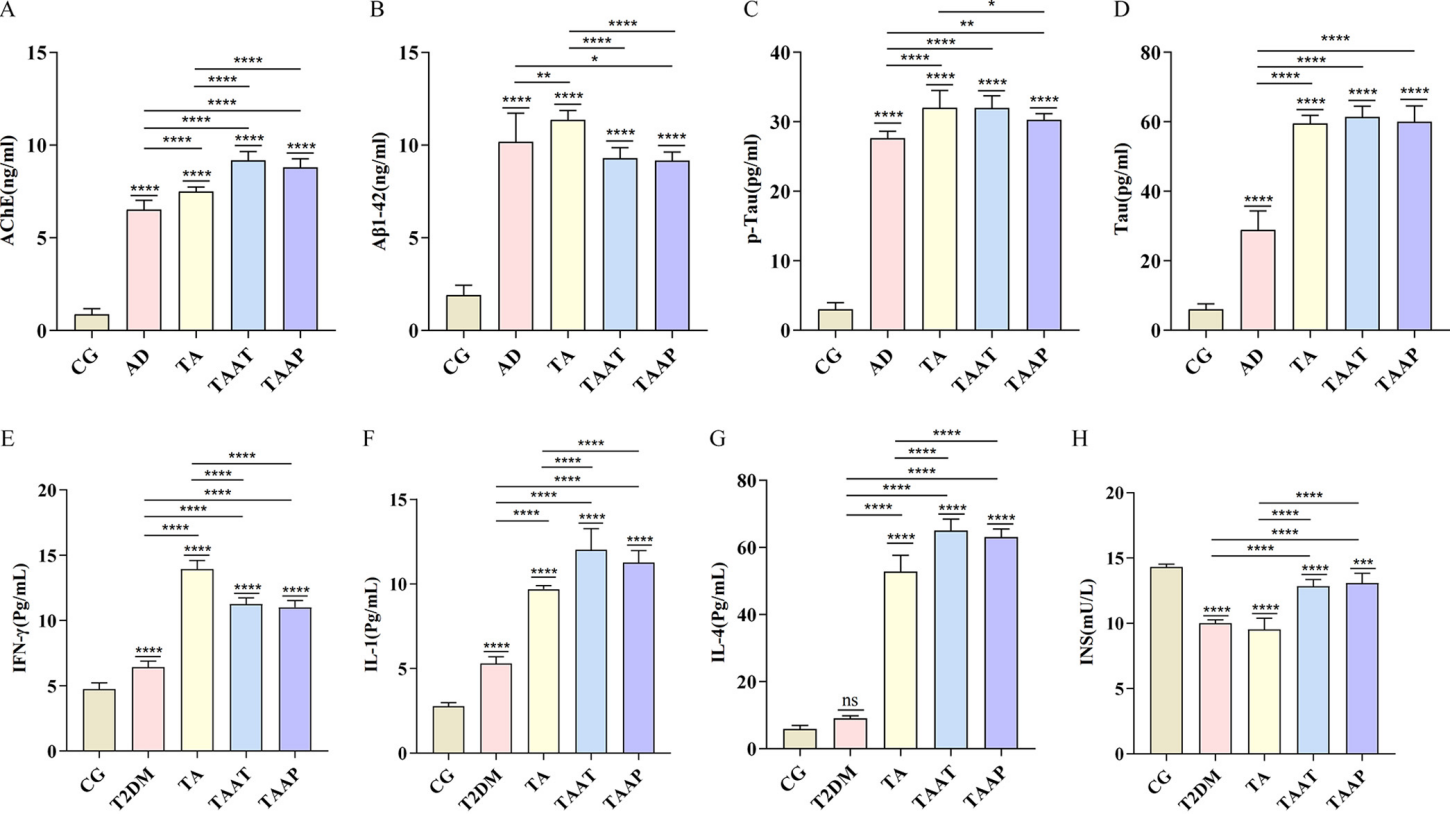
Levels of whole-body tissue inflammation factors and AD factors according to whole-brain tissue examination in different groups of zebrafish. (A) AChE; (B) Aβ1-42; (C) p-Tau; (D) Tau; (E) IFN-γ; (F) IL-4; (G) IL-1; (H) INS. The data are expressed as the mean ± SEM and were analyzed by one-way ANOVA followed by Tukey's *post hoc* test. Significance was defined as follows versus control groups: *, *P* < 0.05; **, *P* < 0.01; ***, *P* < 0.001; and ****, *P* < 0.0001.

Glucose metabolism is impaired and inflammation is aggravated by hyperglycemia in T2DM (19). Body mass index (BMI) is an indicator of diabetes risk (10, 20). Here, we investigated if feeding zebrafish a high-glucose fairy shrimp (HGFS) diet and exposing them to 500 μg/L AlCl_3_·6H_2_O can trigger a combination of T2DM and AD (TA phenotype). This analysis revealed that zebrafish in the T2DM group had higher blood glucose levels (*P* < 0.0001, control group [CG] versus T2DM) (Fig. 2C) and BMI (*P* < 0.0001, CG versus T2DM) (Fig. 2D). Moreover, biochemical markers of T2DM, including triglycerides (TG) (*P* < 0.0001, CG versus T2DM) (Fig. 2E), total cholesterol (T-CHO) (*P* < 0.0001, CG versus T2DM) (Fig. 2G), and low-density lipoprotein (LDL) (*P* = 0.0306, CG versus T2DM) (Fig. 2F) were significantly higher, and there were significant reductions in the levels of high-density lipoprotein (HDL) (*P* = 0.0005, CG versus T2DM) (Fig. 2H) and insulin (INS) (*P* < 0.0001, CG versus T2DM) (Fig. 3H). Additionally, we observed that the T2DM model was associated with inflammation, as revealed by significantly elevated levels of systemic gamma interferon (IFN-γ) in the T2DM group (*P* < 0.0001, CG versus T2DM) (Fig. 3E) and interleukin-1 (IL-1) (*P* < 0.0001, CG versus T2DM) (Fig. 3G) compared to the CG. Together, these data indicated the successful establishment of the zebrafish model of T2DM. AD causes persistent cognitive impairment and is clinically associated with elevated levels of acetylcholinesterase inhibitors (AChE), β-amyloid 1-42 (Aβ1-42), phospho-Tau protein (p-Tau), and Tau protein. Compared with the control group, the zebrafish AD model showed significantly elevated levels of AChE (*P* < 0.0001, CG versus AD) (Fig. 3A), Aβ1-42 (*P* < 0.0001, CG versus AD) (Fig. 3B), p-Tau (*P* < 0.0001, CG versus AD) (Fig. 3C), and Tau (*P* < 0.0001, CG versus AD) (Fig. 3D), indicating the model was successful. Next, we established a zebrafish model of both T2DM and AD (TA). Analysis of the indicators of T2DM and AD showed they were significantly elevated in the TA group compared to the CG. Moreover, the levels of blood glucose (*P* < 0.0001, T2DM versus TA) (Fig. 2C), IFN-γ (*P* < 0.0001, T2DM versus TA) (Fig. 3E), IL-1 (*P* < 0.0001, T2DM versus TA) (Fig. 3G), and IL-4 (*P* < 0.0001, T2DM versus TA) (Fig. 3F) were further elevated compared to those in T2DM zebrafish, indicating the successful establishment of the TA model.

### After pasteurization, the morphological makeup and protein content of *A. muciniphila* were not significantly altered.

**FIG 4:**
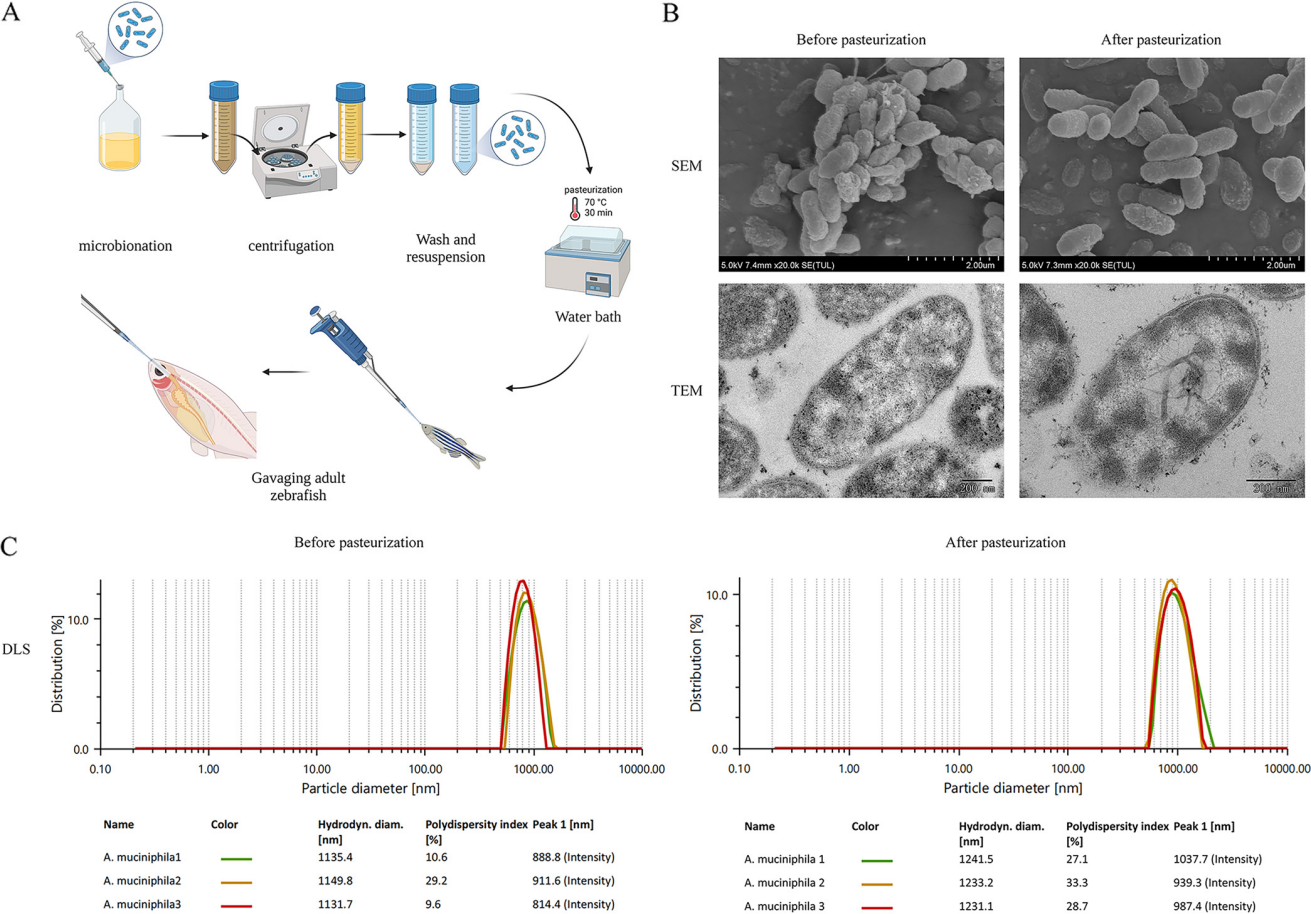
Characteristics of *A. muciniphila* before and after pasteurization. (A) Flow chart of preparation and intragastric administration of bacterial liquid; (B) scanning electron microscope and transmission electron microscope images of *A. muciniphila* before and after pasteurization; (C) dynamic light scattering of *A. muciniphila* before and after pasteurization.

After washing a suspension of *A. muciniphila* three times and pasteurizing it, the measuring dishes were placed in an Anton Paar nanoparticle sizing and zeta potential analyzer for zeta potential and particle size measurement. Measurements were repeated three times. Dynamic light scattering (DLS) analysis showed that *A. muciniphila* expanded upon pasteurization, with the DLS increasing from 1,139.0 nm to 1,235.2 nm. The zeta potential analysis showed that after pasteurization, *A. muciniphila* was more stable and more evenly distributed in phosphate-buffered saline (PBS), with the zeta potential increasing from −15.9 to −17.4 (see Fig. S2 in the supplemental material). These results are consistent with scanning electron microscopy (SEM) and transmission electron microscopy (TEM), which showed that upon pasteurization, thalli expand, and the structure of *A. muciniphila* was generally retained after pasteurization (Fig. 4B). Moreover, SDS-PAGE revealed that the total protein content of *A. muciniphila* was retained after pasteurization and was equivalent to those of unpasteurized samples (Fig. S2). This indicates that pasteurization did not change the protein structure and content and that *A. muciniphila* retained its beneficial effects.

### Blood glucose levels and BMI in TA zebrafish were effectively lowered using pasteurized *A. muciniphila*.

Studies have shown a negative association between *A. muciniphila* content and being overweight, obesity, and untreated T2DM (10) and that *A. muciniphila* improves fat metabolism, glucose tolerance, and IR (21). To assess whether pasteurized *A. muciniphila* could prevent TA, it was used to treat zebrafish during the process of establishing the TA model. To assess if pasteurized *A. muciniphila* could alleviate the symptoms of TA, it was used to treat the established zebrafish model of TA. These analyses showed that treatment of the TAAT group (zebrafish with T2DM plus AD treated with pasteurized *A. muciniphila*) or TAAP group (zebrafish treated with pasteurized *A. muciniphila* for prevention of T2DM plus AD) with pasteurized *A. muciniphila* significantly reduced fasting glucose levels (*P* = 0.1509, CG versus TAAT; *P* = 0.1123, CG versus TAAP) (Fig. 2C) and significantly suppressed BMI increase (*P* = 0.0875, CG versus TAAT; *P* = 0.6615, CG versus TAAP; Fig. 2D). These findings indicate that pasteurized *A. muciniphila* effectively controls blood glucose level and BMI and that it can prevent and improve diabetes mellitus. Analysis of the effects of pasteurized *A. muciniphila* on carbohydrate metabolism in TAAT and TAAP zebrafish revealed that it significantly improved the levels of TG (*P* = 0.0005, CG versus TAAT; *P* < 0.0001, CG versus TAAP) (Fig. 2E), total cholesterol (T-CHO) (*P* < 0.0001, CG versus TAAT; *P* < 0.0001, CG versus TAAP) (Fig. 2G), LDL-cholesterol (LDL-C) (*P* = 0.5850, CG versus TAAT; *P* = 0.9935, CG versus TAAP) (Fig. 2F), HDL-cholesterol (HDL-C) (*P* = 0.0423, CG versus TAAT; *P* = 0.5121, CG versus TAAP) (Fig. 2H) and insulin (*P* < 0.0001, CG versus TAAT; *P* = 0.0001, CG versus TAAP) (Fig. 3H), and that the levels of TG and HDL-C were more effectively improved in TAAP than in TAAT zebrafish. These indicate that pasteurized *A. muciniphila* markedly improves carbohydrate metabolism and that daily pasteurized *A. muciniphila* supplementation can effectively reduce the risk of diabetes.

### Memory, anxiety, aggression, and social preferences were improved in TA zebrafish using pasteurized *A. muciniphila*.

**FIG 5:**
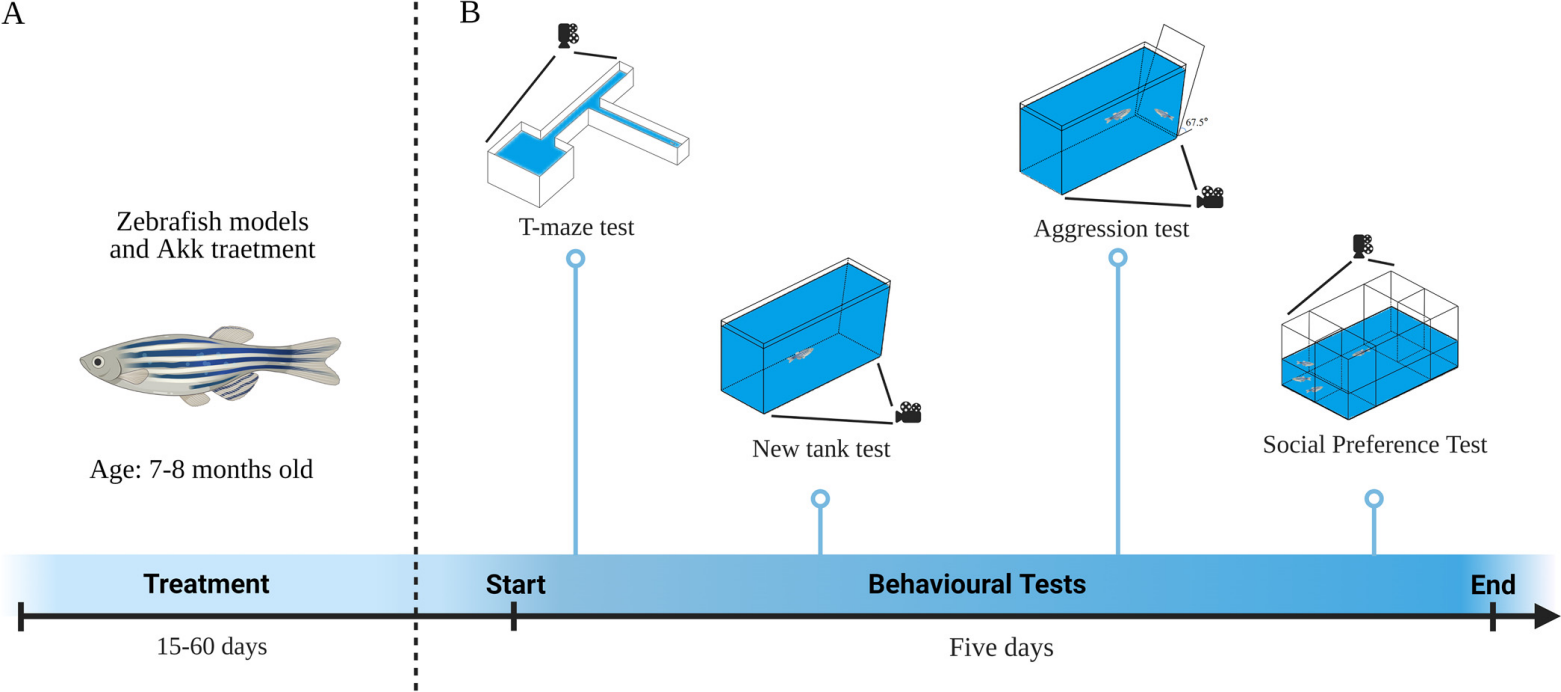
Behavioral tests for zebrafish: timeline. (A) Behavioral tests were performed on zebrafish after model construction and *A. muciniphila* treatment. (B) The memory of zebrafish was tested by T-maze. Then, the anxiety-like behavior of zebrafish was detected by NTT. In addition, the aggressive behavior of zebrafish was determined by aggression test. Finally, the social preference behavior of zebrafish was measured by the social preference test. The whole behavioral test lasted 5 days.

**FIG 6:**
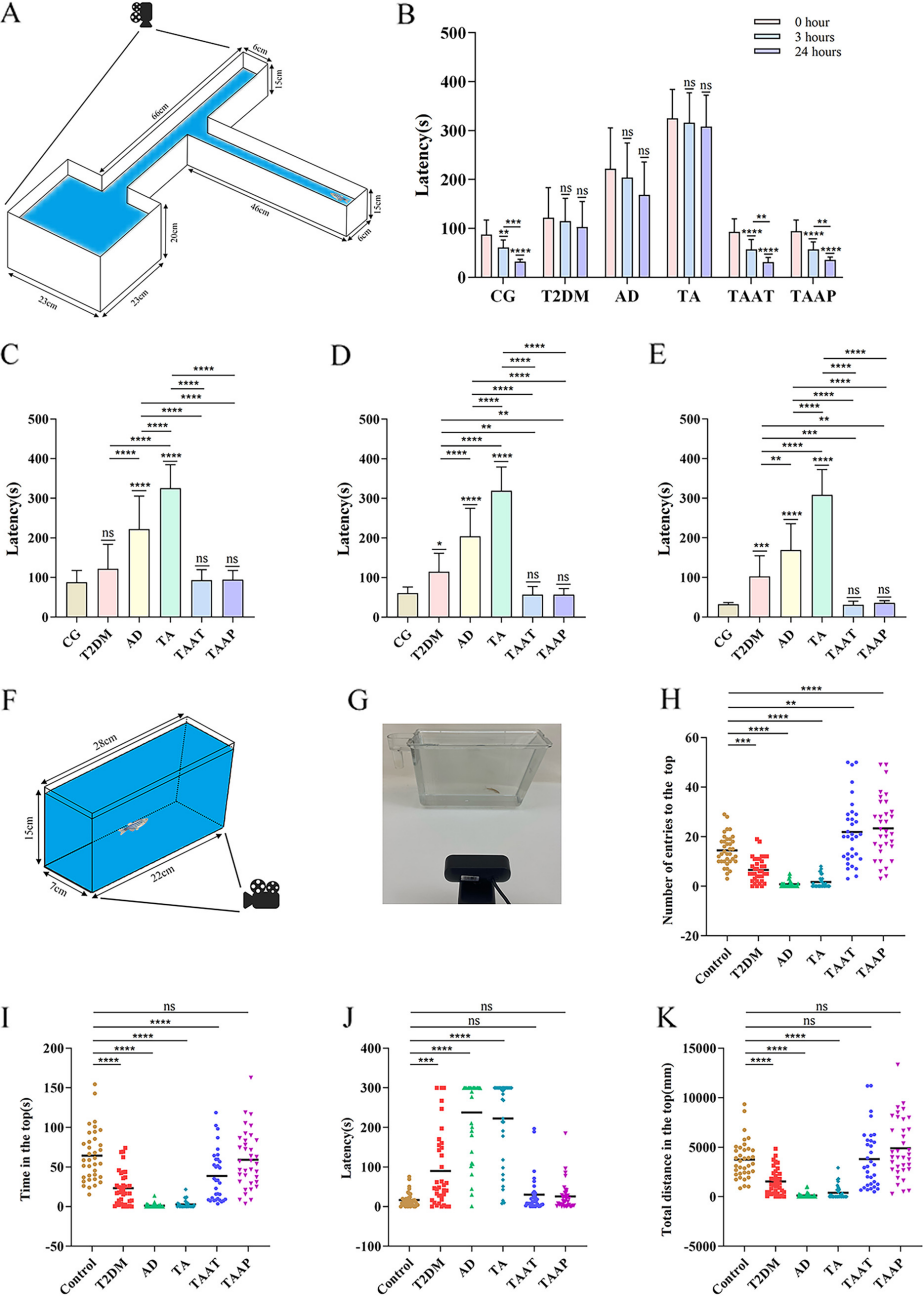
Memory behavior of zebrafish in the T-maze test and anxiety-like behavior of zebrafish in the novel tank test. (A) T-maze schematic; (B) time trend diagram of different groups of zebrafish to find the deep-water area; (C) time it takes the zebrafish to find deep water at 0 h; (D) time it takes the zebrafish to find deep water at 3 h; (E) time it takes the zebrafish to find deep water at 24 h; (F) schematic diagram of NTT; (G) photo of the NTT; (H) number of entries to the top transition in NTT; (I) duration of zebrafish in the top; (J) latency for zebrafish to enter the top; (K) total distance for zebrafish in the top. Numbers of zebrafish: T-maze, *n* = 14 per group; NTT, *n* = 12; CG, T2DM, AD, and TA, *n* = 11 for the TAAT and TAAP groups.

**FIG 7:**
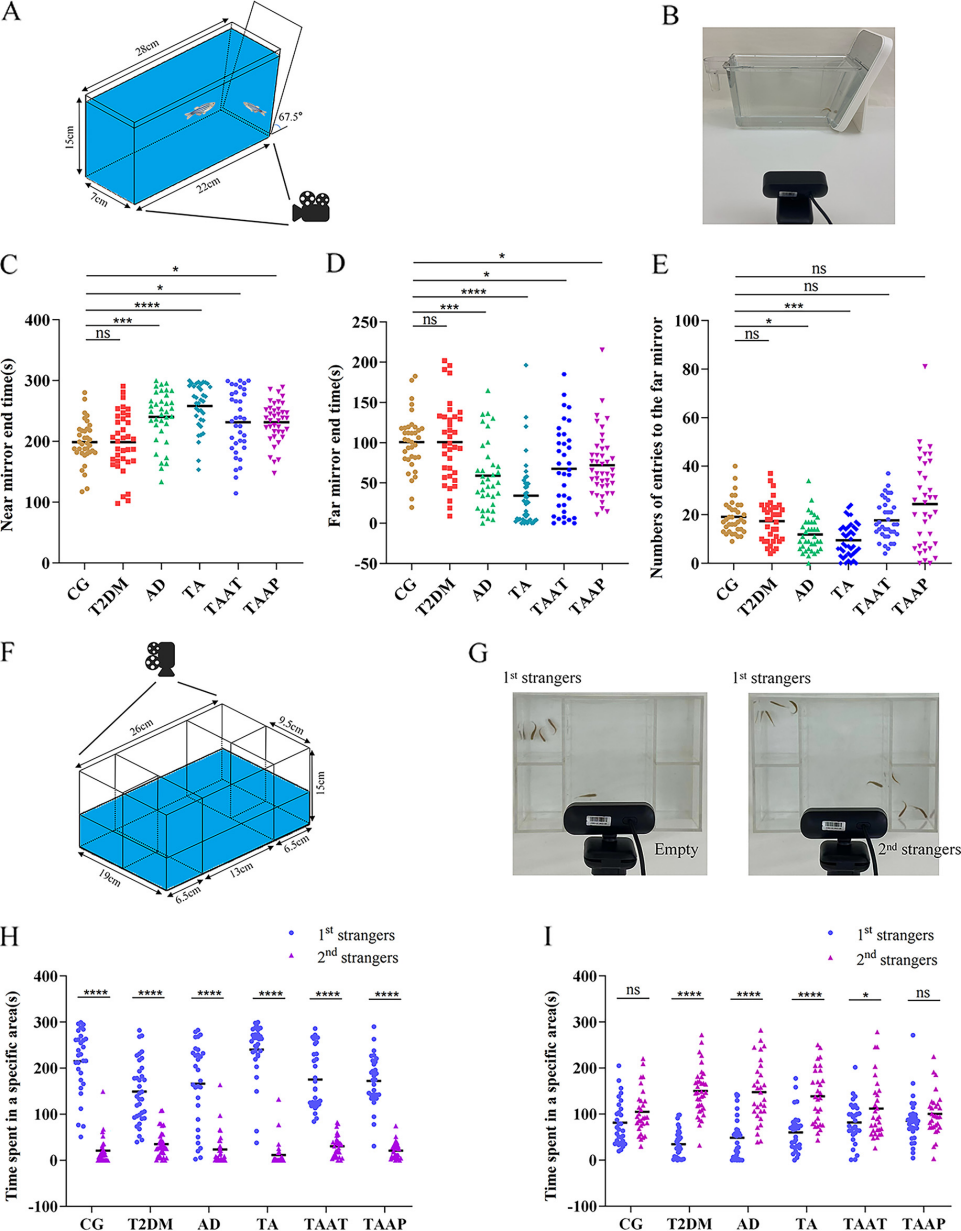
Aggressive behavior of zebrafish in the aggression test and social preference behavior of zebrafish in the social preference test. (A) Schematic diagram of the aggression test; (B) photo of the aggression test; (C) duration of zebrafish near the mirror end; (D) duration of zebrafish at the far mirror end; (E) number of zebrafish entries to the far mirror; (F) schematic diagram of the social preference test; (G) photo of the social preference test; (H) social preference; (I) preference for social novelty. Numbers of zebrafish: aggressive test, *n* = 12 per group; social preference test, *n* = 10 per group.

Behavioral tests were performed on zebrafish immediately after the successful establishment of the model and the end of treatment. The timeline for the zebrafish behavioral tests is shown in Fig. 5. Analysis of T-maze results showed that the time taken by the CG zebrafish to find the enriched deep chamber (EC) region in the T-maze shortened with the increase in the number of experiments. There were significant differences in the times taken to find the EC region between the first, second, and third experiments. However, the time taken by model zebrafish to find the EC region was not shortened with the increase in the number of experiments. Specifically, there was no difference between 0 h versus 3 h (*P* = 0.0022, CG) and 3 h versus 24 h (*P* = 0.009) (Fig. 6B). After feeding on pasteurized *A. muciniphila*, the memory behavior of TAAT and TAAP groups improved significantly at 0 h (*P* = 0.9998, CG versus TAAT; *P* = 0.9994, CG versus TAAP; *P* < 0.0001 TA versus TAAT; *P* < 0.0001 TA versus TAAP) (Fig. 6C), 3 h (*P* = 0.9998, CG versus TAAT; *P* = 0.9998, CG versus TAAP; *P* < 0.0001, TA versus TAAT; *P* < 0.0001, TA versus TAAP) (Fig. 6D) and 24 h (*P* > 0.9999, CG versus TAAT; *P* > 0.9999, CG versus TAAP; *P* < 0.0001 TA versus TAAT; *P* < 0.0001, TA versus TAAP) (Fig. 6E). The time taken to find the EC region gradually became shorter with the increase in experiment times, indicating that pasteurized *A. muciniphila* improved and prevented memory disorders induced by TA treatment. In the new tank test (NTT) experiment, pasteurized *A. muciniphila* alleviated the anxiety-like behavior of TA zebrafish, and the number of entries to the top (*P* = 0.0022, CG versus TAAT; *P* < 0.0001, CG versus TAAP) (Fig. 6H), time in the top (*P* = 0.0006, CG versus TAAT; *P* = 0.9582, CG versus TAAP) (Fig. 6I), latency for zebrafish to enter the top (*P* = 0.9761, CG versus TAAT; *P* = 0.9954, CG versus TAAP) (Fig. 6J), and total distance in the top (*P* > 0.9999, CG versus TAAT; *P* = 0.1349, CG versus TAAP) (Fig. 6K). Notably, the TA group exceeded the CG group in the number of entries to the top. This indicated that pasteurized *A. muciniphila* significantly improved the anxiety-like behavior caused by TA and inhibited the effects of TA (Fig. S3). In the aggression test, pasteurized *A. muciniphila* partially alleviated the TA-induced aggressive behavior, as evidenced by the duration at the near mirror end (*P* = 0.0150, CG versus TAAT; *P* = 0.0123, CG versus TAAP) (Fig. 7C), the duration at the far mirror end (*P* = 0.0182, CG versus TAAT; *P* = 0.0468, CG versus TAAP) (Fig. 7D), and the entry times of the far mirror end (*P* = 0.2818, CG versus TAAT; *P* = 0.9999, CG versus TAAP) (Fig. 7E). The results indicated that pasteurized *A. muciniphila* provided palliative effects in the treatment and prevention of TA-induced aggression (Fig. S3). The social preference test is performed to evaluate social preference behavior. In the present study, all groups of zebrafish remained as 1st strangers for a long time during the first interaction (Fig. 6H). In the second interaction, the duration of zebrafish in the CG group was the same as those in the groups of 1st strangers and 2nd strangers, but the zebrafish in the model group showed a significant difference in the second interaction, which lasts longer in 2nd strangers (*P* = 0.0610, CG; *P* < 0.0001, T2DM; *P* < 0.0001, AD; *P* < 0.0001, TA) (Fig. 7I), indicating that TA induced social preference disorder in zebrafish. Social preference behavior was improved significantly after pasteurization with *A. muciniphila*; the TAAP group showed better outcomes than the TAAT group (TAAT, *P* = 0.0439; TAAP, *P* = 0.2366) (Fig. 7I), and there was no significant difference in the second interaction. Zebrafish social preference behavior was significantly improved by pasteurized *A. muciniphila* (Fig. S4). In summary, these findings demonstrated that pasteurized *A. muciniphila* effectively alleviated anxiety-like behavior, increased aggression, and memory and social preference disorders associated with diabetes and Alzheimer's disease.

### The level of Aβ protein in the brain of TA zebrafish was alleviated with *A. muciniphila*.

The contents of AChE, Aβ1-42, p-Tau, and Tau in the brain of zebrafish were measured after the behavioral tests. It was found that although pasteurized *A. muciniphila* did not reduce AchE (*P* < 0.0001, CG versus TAAT; *P* < 0.0001, CG versus TAAP) (Fig. 3A) and Tau protein (*P* < 0.0001, CG versus TAAT; *P* < 0.0001, CG versus TAAP) (Fig. 3D), it partially reduced Aβ1-42 (*P* < 0.0001, TA versus TAAT; *P* < 0.0001, TA versus TAAP) (Fig. 3B). Although Aβ1-42 in the treatment group was still significantly different from that in the CG group, it was proven that pasteurized *A. muciniphila* had a partial scavenging effect on Aβ1-42. Pasteurized *A. muciniphila* treatment significantly restored the level of HDL-C and HDL-C, and prevented AD-related pathology in the brain. TAAT and TA groups had similar levels of p-Tau, but the content of the p-Tau in TAAP was relatively lower than that in TA group (*P* < 0.0476, TA versus TAAP) (Fig. 3C). p-Tau production was slightly inhibited by pasteurized *A. muciniphila*. In summary, pasteurized *A. muciniphila* restored Aβ1-42 and p-Tau levels in diabetes and Alzheimer's disease.

### Partial immune response in TA zebrafish was induced with pasteurized *A. muciniphila*.

To determine how pasteurized *A. muciniphila* affects TA zebrafish systemic inflammation indexes, further experiments were conducted. Intestinal adaptive immune response is a key determinant of the host’s health, and some intestinal bacteria regulated the generation of the host’s adaptive immune response (22). Here, we found that the IFN-γ level was significantly lower than that of TA group (*P* < 0.0001, TA versus TAAT; *P* < 0.0001, TA versus TAAP) (Fig. 3E), IL-1 (*P* < 0.0001, TA versus TAAT; *P* < 0.0001, TA versus TAAP) (Fig. 3G), IL-4 (*P* < 0.0001, TA versus TAAT; *P* < 0.0001, TA versus TAAP) (Fig. 3F) significantly increased, and IFN-γ was still significantly different compared with the CG group. Moreover, pasteurized *A. muciniphila* induced an adaptive immune response, which increased the *in vivo* expression levels of IL-1 and IL-4. In summary, pasteurized *A. muciniphila* after pasteurization not only relieved TA-induced diabetes indexes and AD indexes but also stimulated an adaptive immune response.

### Intestinal health of TA zebrafish was improved with pasteurized *A. muciniphila*.

**FIG 8:**
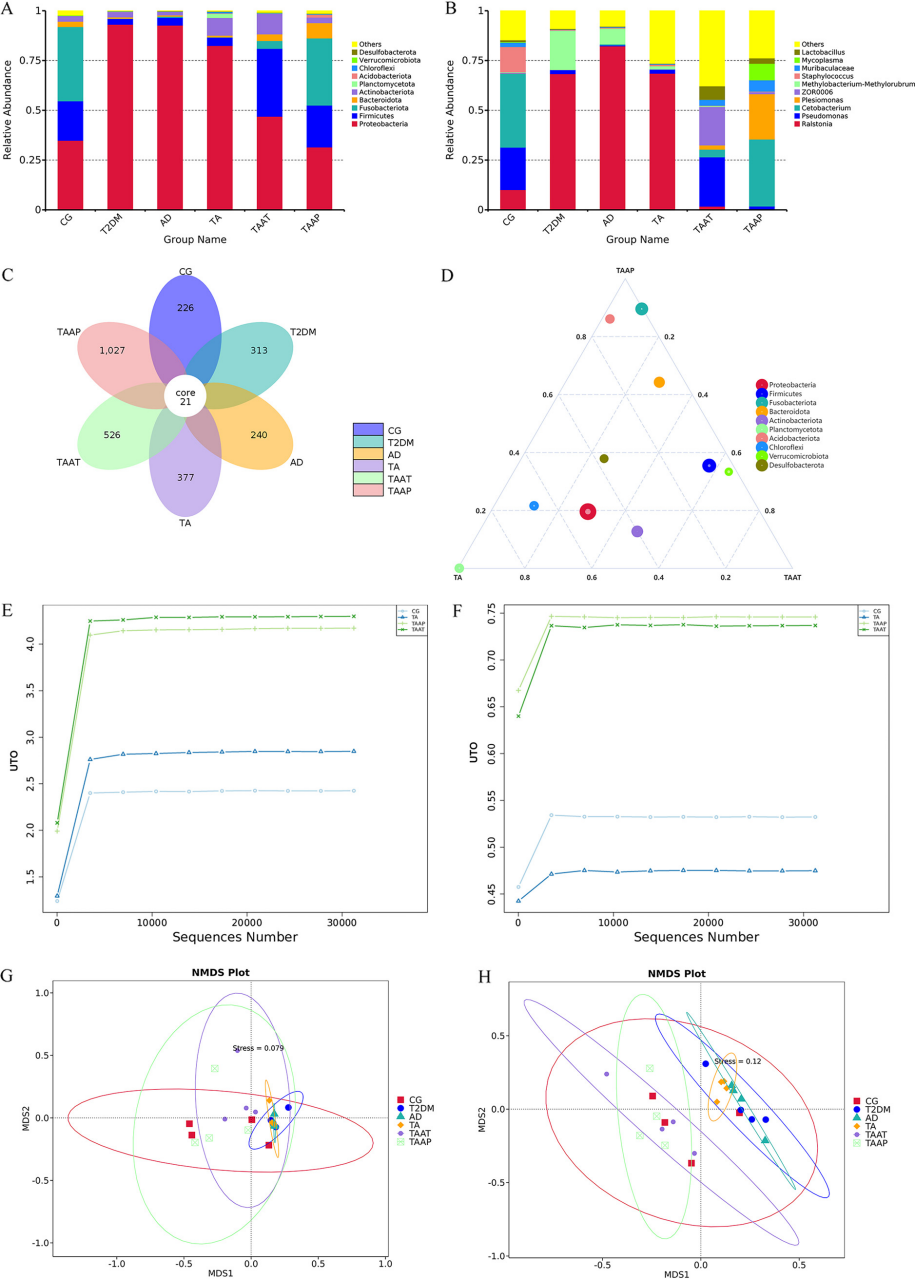
Differential enrichment of intestinal microorganisms in different groups of zebrafish. Distribution of top 10 bacterial taxa averaged at the (A) phylum and (B) genus levels in gut from all groups; (C) flower graph based on operational taxonomic units in the zebrafish intestinal microbiota of all groups; (D) ternary plot graph illustrating the different bacterial species in intestinal samples. (E) The Shannon index was used to determine the community diversity of zebrafish intestines. (F) The Simpson index was used to characterize the diversity and evenness of species distribution in zebrafish intestinal community. (G and H) Weighted and unweighted nonmetric multidimensional scaling (NMDS) was used to measure zebrafish intestinal bacterial composition profile treatment.

Treatment with pasteurized *A. muciniphila* partially restored the richness of intestinal microflora in TA zebrafish. We also found that the health status of intestinal microflora determined the disease progression. Once the zebrafish model was established, we compared the intestinal microflora of T2DM and AD between the TA and CG groups (Fig. 8A and B). At the phylum level, *Proteobacteria* (34.85%), *Firmicutes* (19.84%), and *Fusobacteriota* (37.28%) in the CG group were the dominant bacteria. In comparison, *Proteobacteria* were the dominant bacteria in other model groups (47.6 to 58.28%). Studies have demonstrated that *Proteobacteria* are a potential microbial feature of disease. This was mainly observed in metabolic disorders, inflammation, and even cancer. However, there is evidence that *Proteobacteria* are elevated in DM and AD (23, 24), suggesting that they may be involved in the development of these diseases. At the genus level, the abundance of *Cetobacterium* and Pseudomonas in the T2DM, AD, and TA groups decreased significantly, while *Ralstonia* and other pathogenic bacteria increased significantly. The abundance of the bacteria was improved significantly in the TAAT and TAAP groups. At the phylum level, the level of *Proteobacteria* in TAAT was 46.93%, while its abundance in the TAAP group was recovered to the normal level (31.51%) and was lower by 3.34% than in the CG. At the genus level, Pseudomonas in the TAAT group recovered to the normal level and was higher by 3.41% than in the CG. In the TAAP group, *Cetobacterium* recovered to the normal level whereas *Plesiomonas* richness increased significantly. In CG, T2DM, AD, TA, TAAT, and TAAP zebrafish, the numbers of amplicon sequence variants (ASVs) in the gut microflora were 247, 334, 261, 398, 547, and 1,048, respectively. Notably, the intestinal microbial diversity of the T2DM, AD, and TA groups was slightly higher than that of the CG. The increase in intestinal microbial diversity in the T2DM, AD, and TA groups was due to the increase in pathogenic bacteria, whereas the increase in intestinal microbial diversity in the TAAT and TAAP groups was due to the increase of probiotics (Fig. 8C). In the ternary phase diagram, the number of probiotics such as *Fusobacteriota* and *Bacteroidota* increased, while the number of harmful bacteria such as *Proteobacteria* decreased (Fig. 8D). Analysis of the α diversity (Shannon and Simpson indexes) revealed that both TAAT and TAAP groups had good community diversity after treatment with pasteurized *A. muciniphila* (Fig. 8E) in terms of species distribution diversity and uniformity (Fig. 8F). The results of β-diversity analysis showed that whether weighted (Fig. 8G) or unweighted (Fig. 8H), the differences among T2DM, AD, and TA zebrafish intestinal groups were not significant, and the intestinal microorganisms tended to be unified, indicating the successful establishment of the zebrafish model. However, the intestinal microorganisms of CG, TAAT, and TAAP showed high diversity after pasteurized *A. muciniphila* treatment in zebrafish.

## DISCUSSION

Intestinal microflora is one of the most important factors influencing the host’s health. The composition, activity, and health of intestinal microflora have been linked to the development of many diseases. There is also evidence that *A. muciniphila* abundance is associated with obesity, T2DM-related metabolic diseases, neurodegenerative diseases, and even cancer (25). This suggests that *A. muciniphila* supplementation may provide a novel strategy for preventing and treating diabetes complicated with Alzheimer's disease. 16S rRNA analysis revealed that after supplementation of pasteurized *A. muciniphila* in TA zebrafish, *Proteobacteria* decreased significantly at the phylum level. In the TAAP group, the abundance of *Proteobacteria* was restored to the normal level and was lower by 3.34% compared with CG. *Proteobacteria* include a variety of pathogenic bacteria that can induce low-grade inflammation in patients with diabetes by secreting endotoxins, flagella, and/or other surface components (23, 26). At the genus level, Pseudomonas in the TAAT group recovered to the normal level and was higher by 3.41% than that in the CG. In the TAAP group, *Cetobacterium* abundance was restored to normal levels. *Cetobacterium* is the core bacterium in fish intestines, which produces a large amount of acetate (27). Acetate activates the parasympathetic nervous system, thereby stimulating insulin secretion from the islet β cells (28, 29).

**FIG 9:**
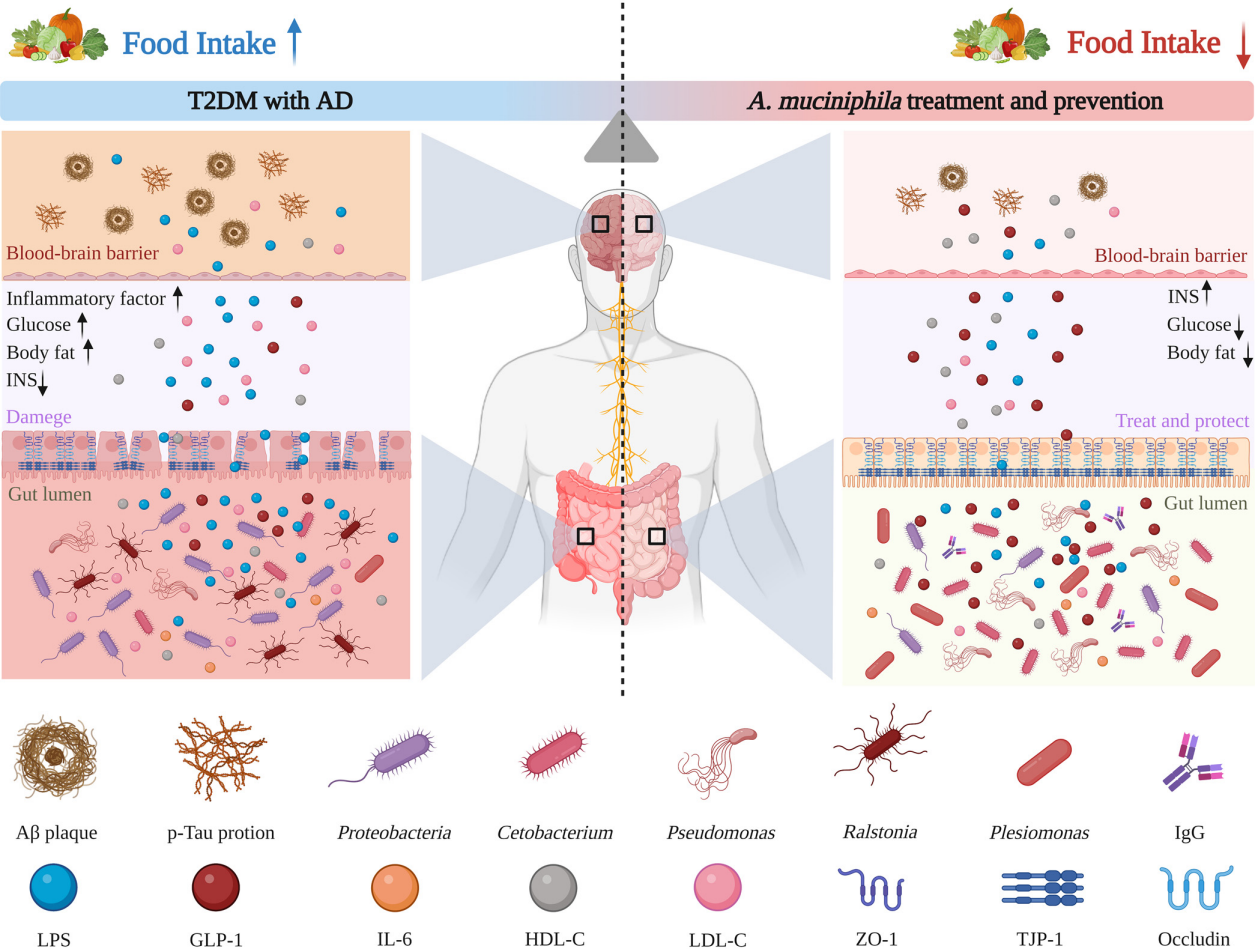
Potential mechanism for using pasteurized *A. muciniphila* to improve health and prevent T2DM with AD.

Treatment with pasteurized *A. muciniphila* significantly improved blood glucose, HDL-C, TG, T-CHO, and LDL-C levels. *A. muciniphila* that has been pasteurized promotes thermogenesis and glucagon-like peptide-1 (GLP-1) release from high-fat diets by enhancing the secretion of uncoupling protein 1 and systemic GLP-1 in brown adipose tissue (30). GLP-1 can promote insulin secretion, control appetite, and promote intestinal mucosal growth, thus improving glucose metabolism and intestinal barrier function (31, 32). Studies have shown that pasteurized *A. muciniphila* also stimulates intestinal cells and macrophages to secrete IL-6. IL-6, in addition to being a proinflammatory factor. *In vitro*, it increased the secretion of GLP-1 in a dose-dependent manner, and stimulated the production of GLP-1 from intestinal endocrine L cells to exert therapeutic effects (30). AD is linked to DM. Patients with AD often show lower levels of HDL-C and higher levels of T-CHO and LDL-C (33, 34). It was found that HDL-C plays a role in the clearance of Aβ protein. Pasteurized *A. muciniphila* treatment restored the level of HDL-C by improving blood and peripheral metabolism, which enhanced Aβ clearance in the brain (9, 35), thus reducing the toxicity of Aβ in the brain. Clinical investigations have reported that patients with T2DM and AD show different degrees of intestinal barrier dysfunction. This intestinal barrier dysfunction causes pathogenic bacteria, lipopolysaccharide, and other metabolites to enter the brain, thereby causing damage. Pasteurized *A. muciniphila* upregulates the expression of tight-junction proteins ZO-1 and TJP-1 and occludin and inhibits the expression of cannabinoid receptor 1 (CB1). Inhibition of CB1 suppresses IR and food intake, which in turn alleviates IR and improves intestinal barrier function (12, 36, 37). In addition, pasteurized *A. muciniphila* was found to induce an immune inflammatory response. Our results showed that pasteurized *A. muciniphila* treatment slightly decreased IFN-γ expression, but increased the levels of the proinflammatory cytokine IL-1 and anti-inflammatory cytokine IL-4. *In vivo*, pasteurized *A. muciniphila* induces antigen-specific T cells and immunoglobulin G1 (IgG1) antibodies, and in the context of intestinal infection or inflammation, it triggers immune inflammatory responses (22). A diagram of the detailed mechanism of pasteurized *A. muciniphila* activity is shown in Fig. 9.

A promising approach integrating behavioral phenotypes with neuropathological features of zebrafish is behavioral phenomics. Behavioral analysis has been widely used to evaluate behavioral abnormalities associated with various diseases. The interaction between internal physiology and external environmental conditions contributes to the formation of animal behavior (38). In this study, we used the T-maze test to evaluate the memory behavior of zebrafish. The results showed that the memory function of TA zebrafish decreased (Fig. 6), which was ascribed to the formation of pathological processes in the brain. Decline in memory function will trigger several problems in patients’ work and life and may be accompanied by depression and other conditions. The occurrence of depression among such patients will result in poor treatment outcomes. A positive and good attitude can improve the self-adaptability of the human body and lessen the severity of diseases. Similarly, memory function plays a very important role in many neurological and metabolic diseases. The memory behavior of zebrafish treated with pasteurized *A. muciniphila* was significantly improved in the TAAT and TAAP groups, indicating that pasteurized *A. muciniphila* enhanced memory and emotion status. The NTT was used to evaluate the anxiety and exercise level of zebrafish. It was observed that the anxiety-like behavior of TA zebrafish increased significantly, whereas the exercise activity level decreased significantly (Fig. 6). The level of exercise activity is a comprehensive expression of cardiovascular, neurological, metabolic, and muscle function. The decline in TA zebrafish behavior indicated impaired physical function as a whole. Notably, treatment with pasteurized *A. muciniphila* improved the anxiety and exercise levels of zebrafish in the TAAT and TAAP groups, indicating that zebrafish can be improved in their overall function by pasteurized *A. muciniphila*.

Furthermore, the aggressive behavior of zebrafish was evaluated with the aggression test. The results showed that the aggressive behavior of TA zebrafish increased significantly (Fig. 7). Aggressive behavior is one of the most common and destructive behaviors of cognitive degradation, which also increases the cost of care for patients and the burden on nurses. The aggressive behavior of zebrafish in the TAAT and TAAP groups was alleviated following treatment with pasteurized *A. muciniphila*. This showed that pasteurized *A. muciniphila* alleviated the aggressive behavior of zebrafish. Social preference test was conducted to evaluate the social preference behavior of zebrafish. The analysis revealed that the social preference behavior of zebrafish in TA group was impaired (Fig. 8). Adult zebrafish, like humans, are social animals that exhibit some group behavior. In this study, the duration of 1st stranger and 2nd strangers of zebrafish in CG group was comparable, but this was not the case for zebrafish in TA group. The preference behavior of TA zebrafish for familiar zebrafish decreased. Following treatment with pasteurized *A. muciniphila*, the social preference behavior of zebrafish in TAAT and TAAP groups was improved, indicating that pasteurized *A. muciniphila* influenced the social preference of zebrafish.

In summary, the present study developed a new type of zebrafish model of diabetes with Alzheimer's disease and explored the effect of pasteurized *A. muciniphila* on diabetes complicated with Alzheimer's disease. Collectively, the results showed that *A. muciniphila* after pasteurization significantly improved and prevented diabetes mellitus complicated with Alzheimer's disease. Pasteurized *A. muciniphila* treatment enhanced the memory, social preference, and aggressive and anxiety behaviors of TA zebrafish and alleviated the pathological features of T2DM and AD. These results open a new window for further development of interventions for people with diabetes complicated with Alzheimer's disease.

## MATERIALS AND METHODS

### Experimental animals.

Wild-type AB zebrafish (Wuhan Zebrafish Resource Center, China) were raised in a standard five-layer single-drainage animal system (Shanghai Haisheng Marine Biological Equipment Co., Ltd., China) with light for 14 h a day and darkness for 10 h, and where system temperature is maintained at 28.0 28.0 ± 1°C. Adult zebrafish 6 to 7 months of age were reared in 12L benchtop aquatic systems (Sensen Marine Biological Equipment Co., Ltd. Jiangsu, China) for 1 month to adapt to the environment. Then they were randomly divided into the following 6 groups (100 strips in each group), each with a with a gender ratio of 1:1: control group (CG), type 2 diabetes mellitus group (T2DM) group, Alzheimer’s disease (AD) group, T2DM-plus-AD (TA) group, *A. muciniphila*-treated T2DM-plus-AD (TAAT) group, and *A. muciniphila*-treated T2DM-plus-AD prevention (TAAP) group.

### Culture and administration of *A. muciniphila.*

*A. muciniphila* (ATCC BAA-835; purchased from Guangdong Microbial Culture Collection Center, Guangzhou, China) was anaerobically cultured at 37°C in fluid thioglycolate medium (no. BNCC153810; purchased from the BeNa Culture Collection, Hebei, China) for 36 h to the logarithmic growth phase. By centrifugation, bacteria were collected and washed three times with aseptic anaerobic PBS. After resuspension in PBS, the optical density at 600 nm (OD_600_) value of bacterial liquid was measured, and the concentration of the bacterial solution was calculated to be 5 × 10^9^ CFU/mL. At the same time, some bacteria were taken to be photographed by scanning electron microscopy (SEM) (SU8020, 5 kV; Japan Hitachi Co., Ltd.), transmission electron microscopy (TEM) (JEM-1200EX, 100 kV; Japan Electronics Co., Ltd.), DLS, and zeta potential (Litesizer 500, Anton Paar Trading Co., Ltd. Shanghai, China) to determine the appearance of bacteria and the dispersion coefficient in PBS. To prepare *A. muciniphila* after pasteurization, the bacterial suspension was heated for 30 min in a water bath at 70°C (12). As described above, pasteurized *A. muciniphila* was taken and photographed by SEM, DLS, and zeta potential to observe changes in the bacterial appearance and dispersion coefficient of pasteurized *A. muciniphila* in PBS.

### Experimental design and sample collection.

Before experiments, all zebrafish were acclimatized in their own tanks for 30 days. Next, we established the zebrafish model of T2DM, as previously described by Wang et al. (39), by continuously feeding the zebrafish high-glucose fairy shrimp for 30 days (see Fig. S1 in the supplemental material). A slight modification was made, based on the methods described by Shang et al. and Senger et al., to configure 500 μg/L AlCl_3_·6H_2_O (Xilong Scientific Co., Ltd., Guangdong, China) solution, adjust the pH to 5.8, change 1/3 of the poison solution every day, and soak for 30 days to build the AD model (40, 41). Based on the diabetes model, a TA model was constructed using the AD model construction method described above. During the establishment of the TA model, the zebrafish were given 5 × 10^9^ CFU/10 μL of pasteurized *A. muciniphila* by intragastric administration with a pipette (TAAP) daily for 60 days. After establishment, the model was administered 5 × 10^9^ CFU/10 μL of pasteurized *A. muciniphila* (TAAT) daily for 60 days. All models underwent behavioral testing, followed by body length and weight measurements for body mass index (BMI) calculation. BMI was calculated using the formula body weight (g)/body length (m) squared. For future analysis, blood glucose levels were determined (39), and we collected intestine tissue, brain tissue, and whole-body samples, and stored them at 80°C.

### T-maze test.

Because patients with T2DM and AD often show memory impairment, we examined the memories of the zebrafish models using the T-maze test (Fig. 6A). The T-maze is made of a long, straight arm divided into two shorter arms connected at 90° angles and a slightly deeper deep-water area filled with marbles and food at the end of one of the short arms. Zebrafish memory was evaluated as described by Darland et al. (42), with slight modifications. The arms in the deep-water area were black, while the other arms were white (42, 43). The amount of time taken by the zebrafish to enter the deep-water area for the first time and stay for at least 20 s was recorded. The test time was 6 min. Memory tests were done at 0 h, 3 h, and 24 h, respectively. As the number of tests increased, the time the zebrafish took to find the deep-water area gradually shortened.

### NTT.

Anxiety is often observed in patients with T2DM and AD. To assess zebrafish anxiety-like behavior, we used the NTT (44) (Fig. 6A; see Video S1 in the supplemental material). To assess NTT behavior, the tank is divided into upper and lower parts. Studies have shown that zebrafish with anxiety-like behavior mostly occupy the lower half. Thus, to assess anxiety-like behavior, we recorded the number of times the zebrafish entered the upper half, their duration of stay in the upper half (seconds), the duration of delay in entering the upper half (seconds), and the total distance (centimeters) covered. The test was done for 5 min. The experiment was repeated three times.

### Aggression test.

People with AD tend to be irritable, lose their temper, and tend to be aggressive. To assess aggressive behavior in the zebrafish model of AD, we used the aggression test (Fig. 7A; Video S2) based on a trapezoidal water tank as described by Gerlai et al., with slight modifications (45). The trapezoidal tank had a mirror (tilted at a 22.5° angle) on the inclined side. Because zebrafish do not recognize themselves in mirrors, seeing their reflections in the mirror triggers aggressive behavior. We recorded the duration of zebrafish behavior at the near and far mirror ends as well as their number of entries into the far mirror end for 5 min in order to assess aggressive behavior (45, 46). The experiment was repeated three times.

### Social preference test.

Because patients with T2DM and AD exhibit social preference disorders (47, 48), we used the social preference test to assess social preference in zebrafish (49) (Fig. 8A; Videos S3 and S4). The water tank used in this experiment is composed of transparent plastic material and divided into five parts, including the central area (13 by 19 cm) and four small areas (6.5 by 9 cm). Water and odor can flow through the walls of the central area and surrounding small areas, which are pierced with 2-mm holes. The experiment is divided into two stages. In the first experiment (social preference test 1), 5 min was allowed for a zebrafish to interact with three unfamiliar wild-type zebrafish (the 1st strangers) in the central area. In the second experiment (social preference test 2), three unfamiliar wild-type zebrafish (the 2nd strangers) were placed in a small area diagonally from the three wild-type zebrafish in the first experiment and recorded for 5 min. In order to evaluate the interaction between the zebrafish in the central area and the other four small areas, the central area is divided into four equal parts, and the residence time of the zebrafish in each area is recorded. Zebrafish social preferences were then assessed by comparing the area closest to the 1st stranger group and the diagonal blank area with the area closest to the 2nd stranger group. In order to reduce the difference caused by sexual attraction, each group of strange fish included two males and one female, and the experiment was repeated three times. Two different cameras were used to capture all behavioral test observations, and FishTrack (XinRuan Information Technology Co., Ltd., Shanghai, China) was used to analyze the results.

### Whole-body and brain tissue biochemical analyses.

The blood glucose level was measured by a micro-glucose meter (Sinocare, Co., Ltd. Changsha, China). First, a blood collection belt (Sinocare, Co., Ltd. Changsha, China) was prepared and inserted into the glucose meter. In order to obtain blood, the method of Gabriela et al. was used to cut a wound obliquely between the caudal fin and the anus (50), after which the blood strip was quickly immersed in the blood, showing blood sugar levels 4 to 8 s later. At the end of BMI and blood glucose tests, the zebrafish were decapitated and brain, intestinal, and body tissues were collected. The brain tissue was homogenized in 0.9% PBS (1:9 [wt/wt]) and centrifuged at 5,000 × *g* at 4°C. The supernatant was collected for biochemical determination. Headless body tissue (whole-body samples) was homogenized in 0.9% saline or ethanol (1:9 [wt/wt]) and centrifuged at 2,500 or 3,000 rpm at 4°C for 10 min. A biochemical detection kit (Nanjing Jiancheng Bioengineering Institute, Nanjing, China) was used to determine high-density lipoprotein cholesterol (HDL-C), triglyceride (TG), total cholesterol (T-CHO) and low-density lipoprotein cholesterol (LDL-C) in whole-body samples. The levels of Aβ1-42, phospho-Tau (p-Tau), Tau, and acetylcholinesterase (AChE) in brain tissue (Shanghai Mlbio Biotechnology Co., Ltd. Shanghai, China) and the levels of IL-1, IL-4, IFN-γ and INS in whole-body samples (Nanjing Jiancheng Biological Engineering Research Institute, Nanjing, China) were detected by an enzyme-linked immunosorbent assay (ELISA) kit.

### 16S rRNA sequencing and analysis.

To assess differences in the gut flora of the different model groups and after treatment, four samples were collected separately per group, with 15 intestines in each sample, and submitted to Novogene Bioinformatics Technology Co., Ltd. (Beijing, China), for analysis. Total bacterial DNA was extracted and sequenced using a standard protocol (51). Briefly, The V3-V4 fragment of the 16S rRNA gene was amplified by primers 341F (5′-CCTAYGGGRBGCASCAG-3′) and 806R (5′-GGACTACHVGGGTWTCTAAT-3′). Next, the library was constructed using a NEBNext Ultra IIDNA library prep kit (Illumina, USA) and quantified using Qubit and quantitative PCR (qPCR), followed by sequencing on an Illumina NovaSeq 6000 platform. After raw sequencing data splicing, quality filtering, and chimera removal, amplicon sequence variants (ASVs) were generated using divisive amplicon denoising algorithm 2. Then classify-sklearn in QIIME2 (Quantitative Insights into Microbial Ecology 2) was used to classify each ASV and calculate the α diversity index and β diversity.

### Statistical analysis.

The statistical analysis was conducted with GraphPad Prism 8 (GraphPad Software, Inc. San Diego, CA, USA). Standard errors of the mean (SEM) of all data are expressed as group mean ± SEM. To compare different groups following the two-tailed unpaired *t* test and one-way analysis of variance (ANOVA), Tukey's *post hoc* test or Dunnett's multiple-comparison test was used. The symbols *, **, ***, and **** indicate *P* values of <0.05, <0.01, <0.001, and <0.0001, respectively. The experiments were conducted by three different researchers in three independent replications.

### Ethics approval and consent to participate.

All animal experiments were approved the Committee on the Ethics of Animal Experiments of Wenzhou University (permission no. WZU-2022-106).

### Data availability.

The data sets used and/or analyzed during the current study are available from the corresponding author on reasonable request.
