# Supplementary material for: Improvement in Zebrafish with Diabetes and Alzheimer's Disease Treated with Pasteurized Akkermansia muciniphila
Source: Microbiol Spectr. 2023 May 16;11(3):e00849-23. doi: 10.1128/spectrum.00849-23 (PMC10269592; doi:10.1128/spectrum.00849-23)
Supplement: Supplemental file 1 — Supplemental material. Download spectrum.00849-23-s0001.docx, DOCX file, 1.5 MB [file spectrum.00849-23-s0001.docx]

Improvement of pasteurized *Akkermansia muciniphila* on zebrafish with diabetes and Alzheimer's disease

**Linkai Qu ^a, b^, Fan Liu ^a^, Yimeng Fang ^a^, Lei Wang ^a^, Haojie Chen ^a^, Qinsi Yang ^c^, Hao Dong ^b^, Libo Jin ^a^ , Wei Wu ^d^ , Da Sun ^a^**

^a^ Institute of Life Sciences & Biomedical Collaborative Innovation Center of Zhejiang Province, Wenzhou University, Wenzhou 325000, China

^b^ College of Life Sciences, Jilin Agricultural University , Changchun 130118, China

^c^ Wenzhou Institute, University of Chinese Academy of Sciences, Wenzhou 325000, China

^d^ Key Laboratory for Biorheological Science and Technology of Ministry of Education, State and Local Joint Engineering Laboratory for Vascular Implants, Bioengineering College of Chongqing University, Chongqing 400030, China

† These authors contributed equally to this work.

*** Correspondence:**

Da Sun, [sunday@wzu.edu.cn](mailto:sunday@wzu.edu.cn), Institute of Life Sciences & Biomedical Collaborative Innovation Center of Zhejiang Province, Wenzhou University, Wenzhou 325000, China;

Wei Wu, david2015@cqu.edu.cn, Key Laboratory for Biorheological Science and Technology of Ministry of Education, State and Local Joint Engineering Laboratory for Vascular Implants, Bioengineering College of Chongqing University, Chongqing 400030, China;

Libo Jin, 20160121@wzu.edu.cn, Institute of Life Sciences & Biomedical Collaborative Innovation Center of Zhejiang Province, Wenzhou University, Wenzhou 325000, China.

**Captions of figures and videos:**

**Figure S1.** Changes of morphological characteristics of fairy shrimps before and after glucose treatment. (A) 24 hours after ddH2O incubated fairy shrimps, fairy shrimps' lyophilized morphological structure. (B) After 20% glucose treatment, the morphological structure of fairy shrimps did not change.

**Figure S2.** Zeta potential and total protein of *A. muciniphila* before and after pasteurization. (A) After pasteurization, the zeta potential of *A. muciniphila* increased slightly, and the dispersion was better. (B) The total protein of *A. muciniphila* after pasteurization was the same as that before pasteurization, and there was no significant difference.

**Figure S3.** Trajectory map and Heat map of zebrafish in novel tank test and aggression test. (A) After *A. muciniphila* treatment, the anxiety-like behavior of zebrafish was significantly improved and there was no significant difference between the control group and the control group in novel tank test. (B) The aggressive behavior of zebrafish treated with *A. muciniphila* was significantly improved compared with that of TA group in aggression test.

**Figure S4.** Trajectory map and Heat map of zebrafish in social preference test. (A) During the first interaction, all groups of zebrafish lasted in 1^st^ strangers for a long time in social preference test 1. (B) In the second interaction, zebrafish in *A. muciniphila* treatment group had the same duration of 1^st^ strangers and 2^nd^ strangers, which was significantly different from that in TA group.

**Video S1.** Schematic diagram of novel tank test (NTT).

**Video S2.** Schematic diagram of aggression test.

**Video S3.** Schematic diagram of social preference test 1.

**Video S4.** Schematic diagram of social preference test 2.

**
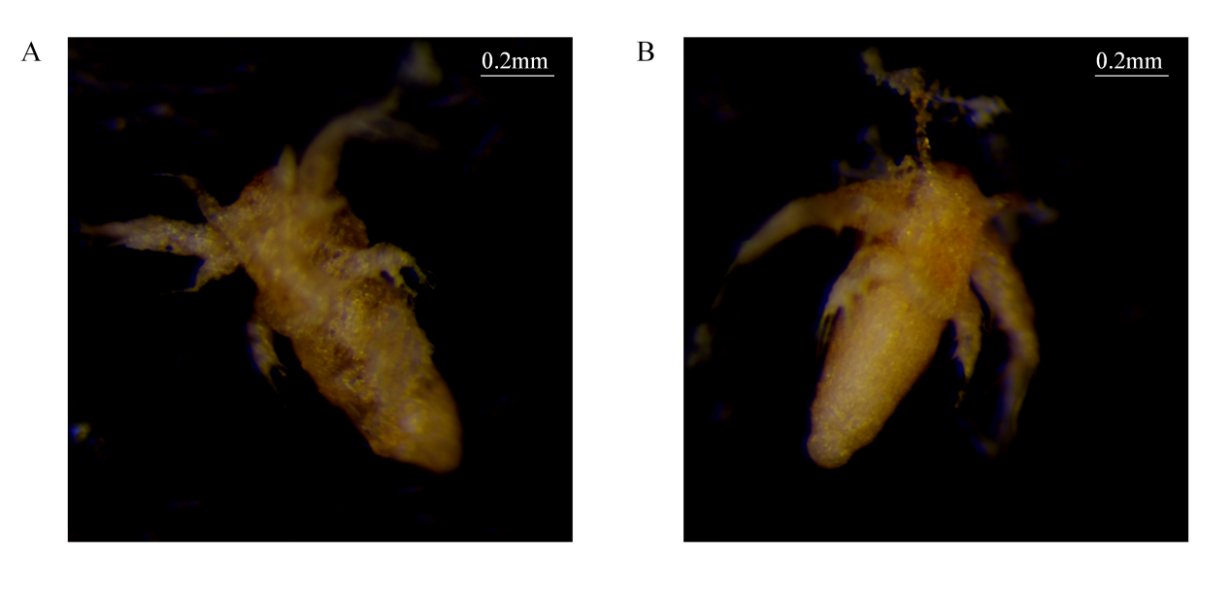
**

**Figure S1.** Changes of morphological characteristics of fairy shrimps before and after glucose treatment. (A) 24 hours after ddH2O incubated fairy shrimps, fairy shrimps' lyophilized morphological structure. (B) After 20% glucose treatment, the morphological structure of fairy shrimps did not change.


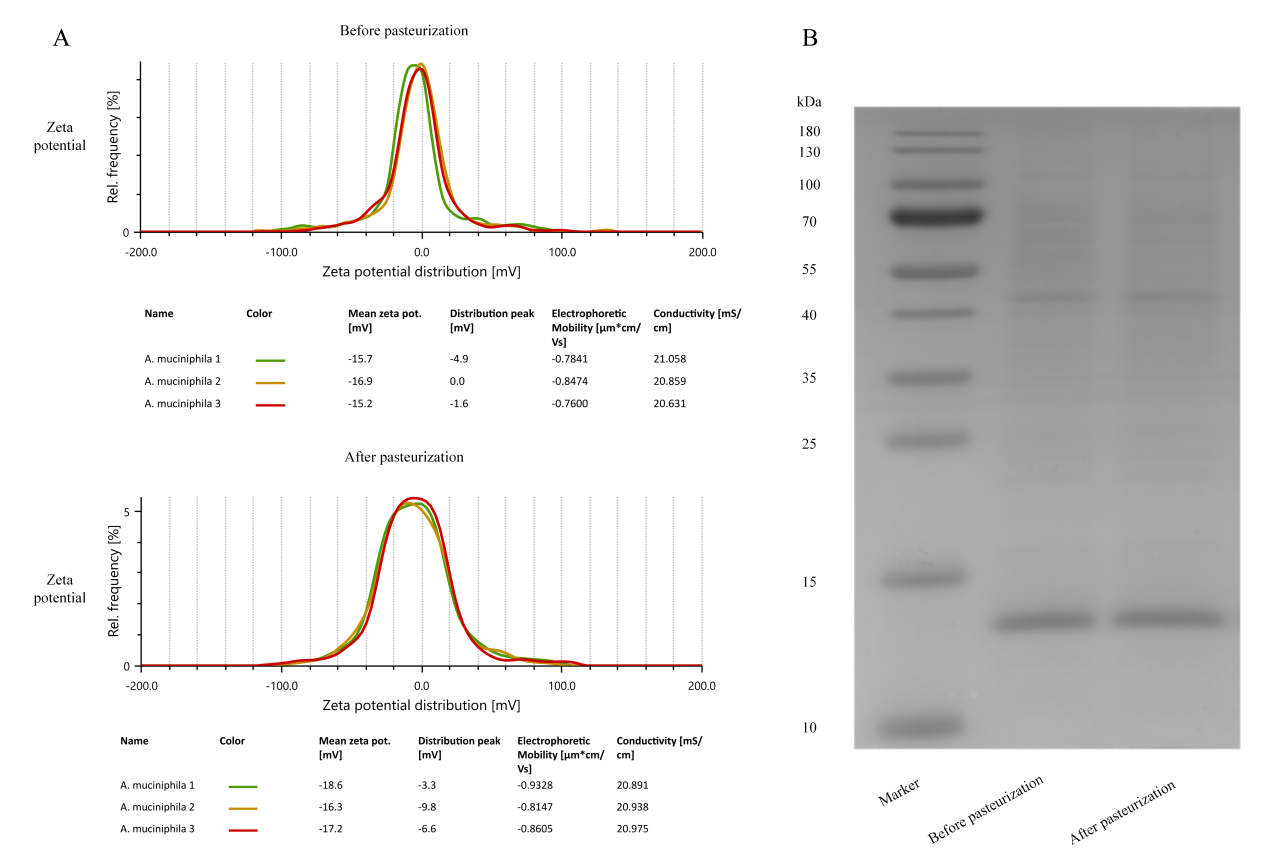


**Figure S2.** Zeta potential and total protein of *A. muciniphila* before and after pasteurization. (A) After pasteurization, the zeta potential of *A. muciniphila* increased slightly, and the dispersion was better. (B) The total protein of *A. muciniphila* after pasteurization was the same as that before pasteurization, and there was no significant difference.


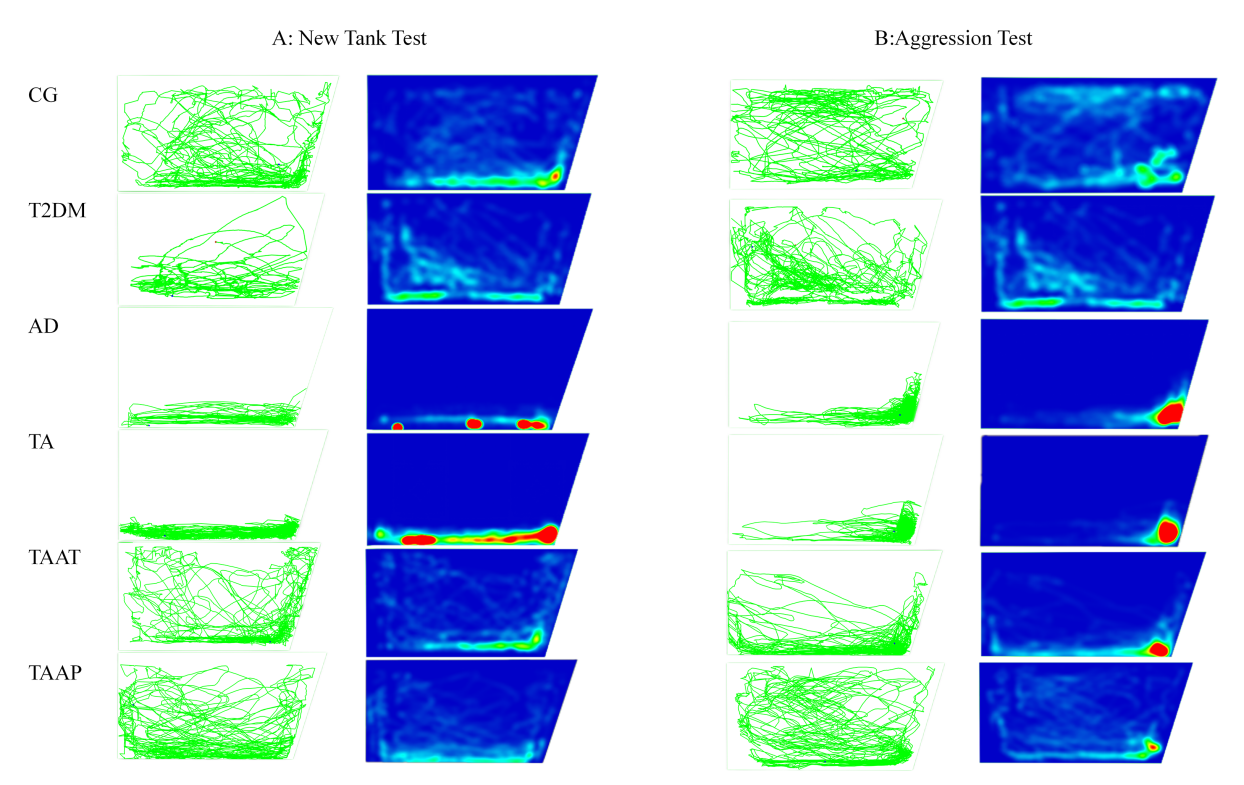


**Figure S3.** Trajectory map and Heat map of zebrafish in novel tank test and aggression test. (A) After *A. muciniphila* treatment, the anxiety-like behavior of zebrafish was significantly improved and there was no significant difference between the control group and the control group in novel tank test. (B) The aggressive behavior of zebrafish treated with *A. muciniphila* was significantly improved compared with that of TA group in aggression test.


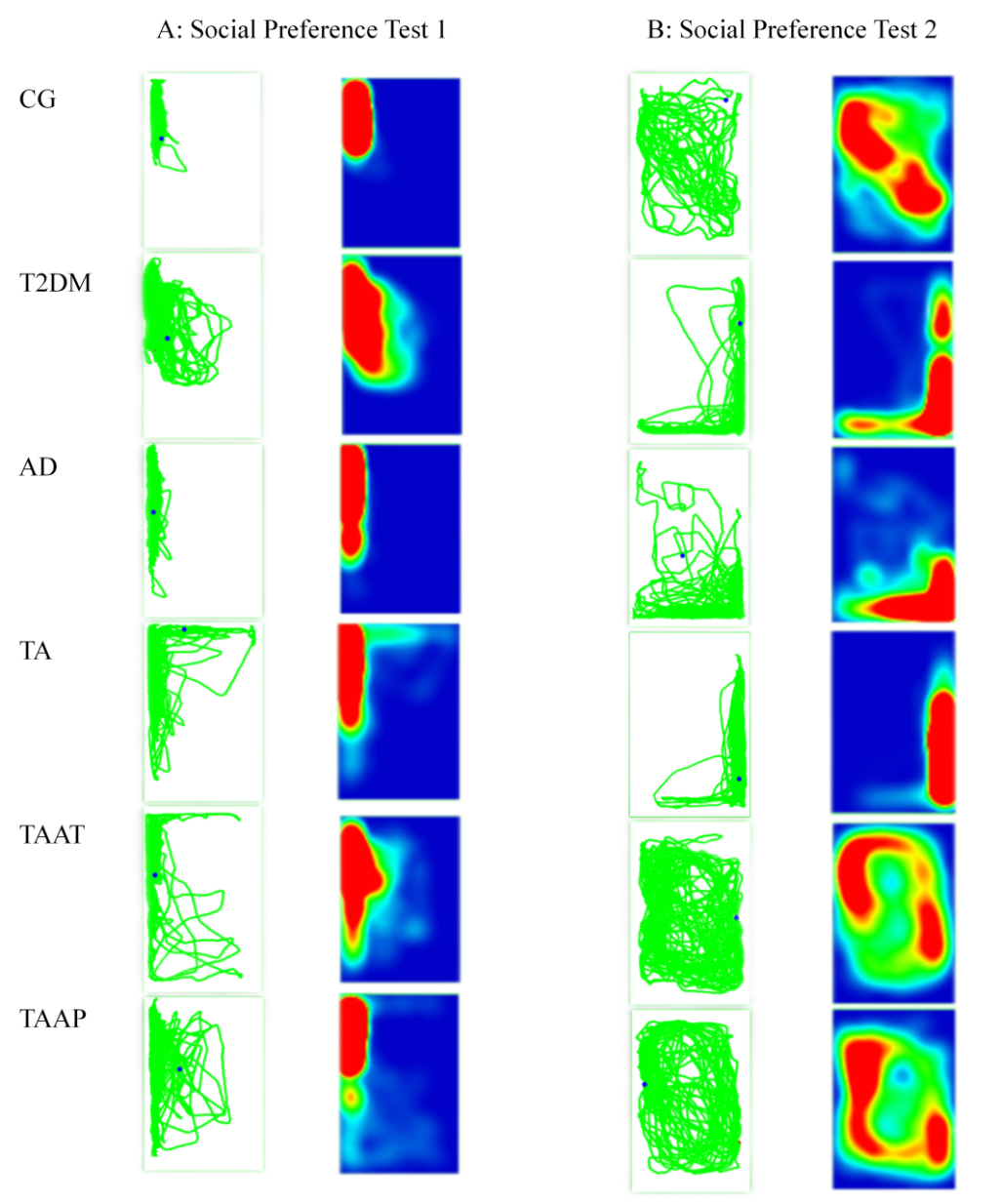


**Figure S4.** Trajectory map and Heat map of zebrafish in social preference test. (A) During the first interaction, all groups of zebrafish lasted in 1^st^ strangers for a long time in social preference test 1. (B) In the second interaction, zebrafish in *A. muciniphila* treatment group had the same duration of 1^st^ strangers and 2^nd^ strangers, which was significantly different from that in TA group.
